# An Unusual Presentation of Paroxysmal Atrial Fibrillation Following Ablation

**DOI:** 10.7759/cureus.27171

**Published:** 2022-07-23

**Authors:** Zachary P Kerosky, Emily Strickland, Takor Arreymbi, Joel Abbott

**Affiliations:** 1 Internal Medicine, Madigan Army Medical Center, Tacoma, USA; 2 Cardiology, Madigan Army Medical Center, Tacoma, USA

**Keywords:** β-blocker, catecholamines, ablation, atrial fibrillation, pheochromocytoma

## Abstract

A pheochromocytoma is a rare catecholamine-secreting tumor with an incidence of 0.8 per 100,000 person-years. Classic clinical manifestations include episodic headache, sweating, and tachycardia. This case report shares a unique presentation in which a patient with a history of atrial fibrillation status post-ablation procedure was admitted for chest pain and found to have imaging and laboratory findings consistent with pheochromocytoma. This case illustrates the importance of a high clinical index of suspicion for a pheochromocytoma since it can have a variety of clinical presentations and can result in unnecessary procedures.

## Introduction

A pheochromocytoma is a rare catecholamine-secreting tumor with an incidence of 0.8 per 100,000 person-years. Classic clinical manifestations include episodic headache, sweating, and tachycardia. Rarely, a pheochromocytoma can be associated with cardiomyopathy secondary to catecholamine excess with significant elevations in initial troponins as well as brain natriuretic peptide, which can result in supraventricular tachyarrhythmias, including atrial fibrillation [1,2].

## Case presentation

**Figure 1:**
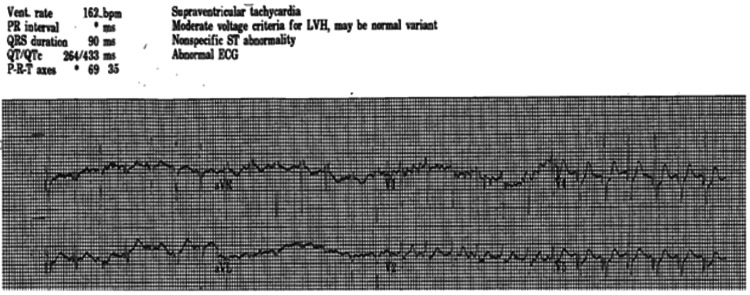
ECG showing supraventricular tachycardia.

A 27-year-old active-duty male soldier with a history of atrial fibrillation status post-ablation procedure four days prior was admitted for chest pain and headache associated with a heart rate of 150 beats per minute and systolic blood pressure of 240 mmHg. He had experienced these symptoms periodically over the last eight months without an apparent trigger. His daily medications included diltiazem, metoprolol, and apixaban. ECG showed supraventricular tachycardia refractory to vagal maneuvers (Figure 1), and he was given adenosine with resolution of the arrhythmia.

**Figure 2:**
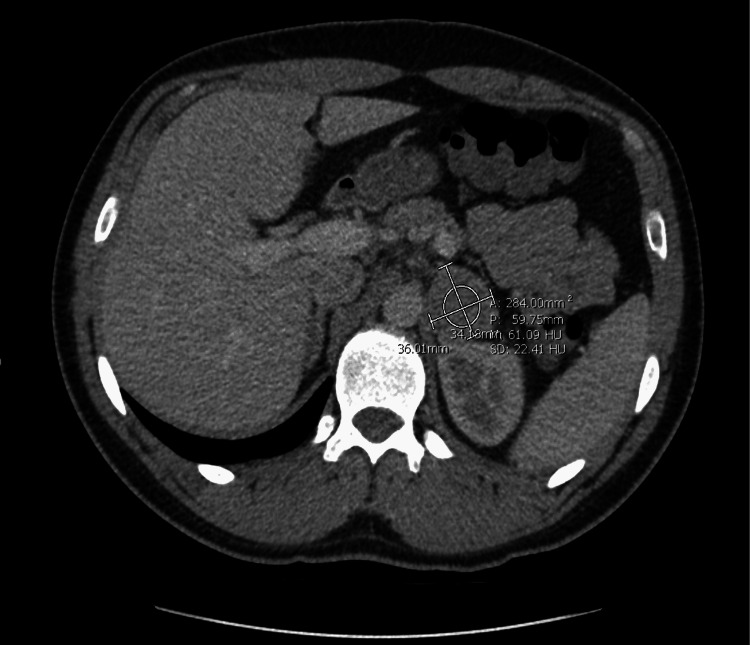
CT adrenal with and without contrast showing a 3.6-cm left-sided adrenal mass.

The initial workup included a negative infectious and neurologic workup, a normal TSH, and a negative urine drug screen. Initial high-sensitive troponin was found to be elevated at 347 ng/L and 367 ng/L with elevation in BNP to 3,089 pg/mL. The diagnostic testing performed included an unremarkable transthoracic echocardiogram. CT chest angiogram was negative for any acute pathology; however, an incidental 3.6-cm left-sided adrenal mass was discovered (Figure 2) [3-6].

Given the constellation of symptoms and unrevealing clinical and initial laboratory findings, pheochromocytoma was suspected [7]. Serum metanephrines and normetanephrines were found to be elevated at 201 pg/mL and 1,924 pg/mL, respectively. CT adrenal study was equivocal for a demonstration of pheochromocytoma but confirmed the presence of adenoma. Repeat serum metanephrines and normetanephrines were drawn, as well as 24-hour fractionated urinary metanephrines and catecholamines, which were found to be consistent with pheochromocytoma. Given the concern for unopposed α-stimulation, his metoprolol was held, and he was subsequently started on phenoxybenzamine that was titrated up to establish α-blockade prior to the reinitiation of his β-blocker.

**Figure 3:**
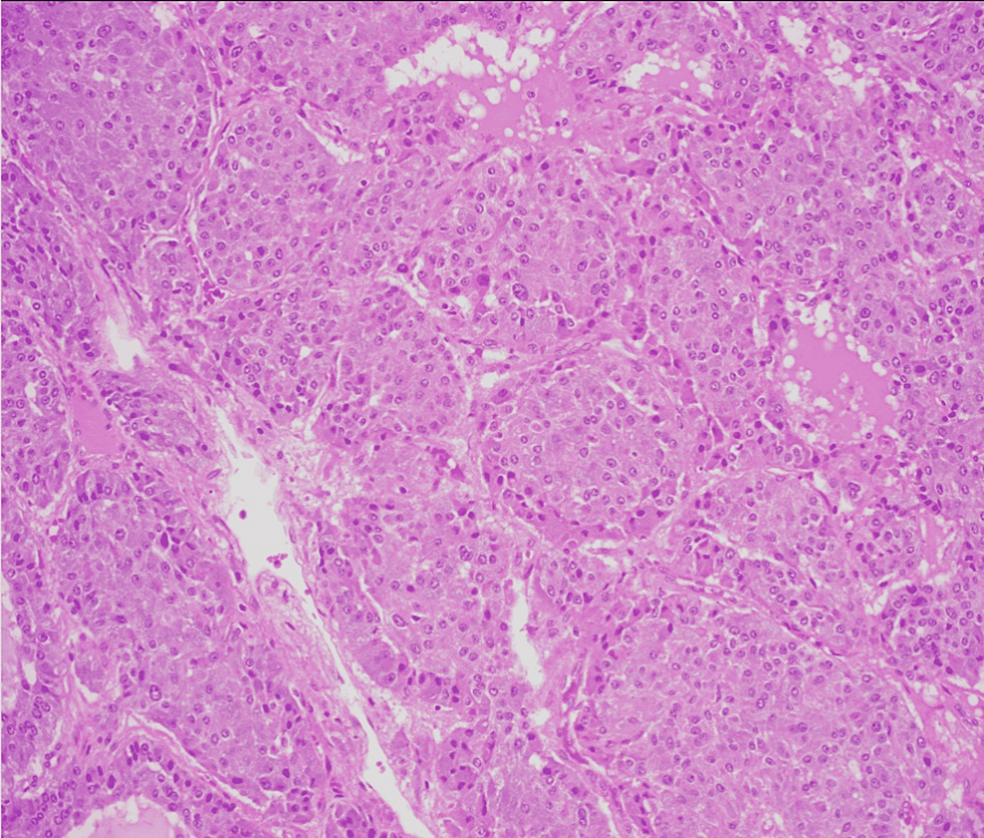
Histology showing polygonal epithelioid cells in compact cell nests.

**Figure 4:**
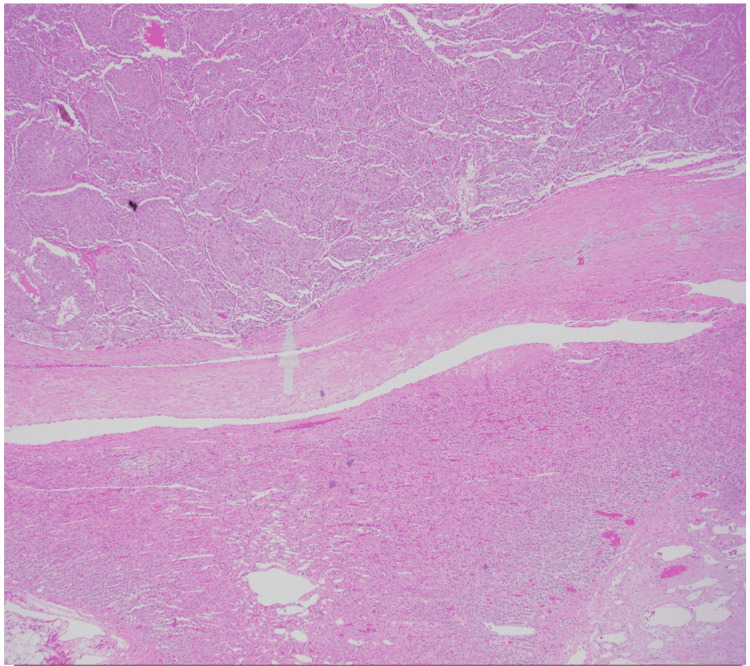
Spindle-shaped sustentacular cells (arrow).

Ultimately, surgery was consulted with surgical removal of the mass. Postoperative hypotension was avoided with adequate fluid replacement. Pathology was completed with histology demonstrating polygonal epithelioid cells in compact cell nests and spindle-shaped sustentacular cells consistent with the diagnosis of pheochromocytoma (Figure 3 and Figure 4). Subsequent genetic testing was performed postoperatively confirming positivity.

## Discussion

Pheochromocytomas produce catecholamines in varying amounts with either continuous or episodic release, which leads to a wide range of clinical manifestations beyond the classical presentation with episodic headache, sweating, and tachycardia [8]. These tumors are rare with an annual incidence estimated at around 0.8 per 100,000 person-years [9]. Pheochromocytomas are well known to carry a high risk of morbidity and mortality with cardiovascular manifestations accounting for around 70% of the mortality [8]. In addition, studies have shown that catecholamines have direct myocardial toxicity that may be long-lasting [2,10]. Quick diagnosis and definitive treatment with surgical removal are important; however, recognition and accurate diagnosis may be challenging given its clinical manifestation variety and rare nature.

Arrhythmias are seen in around 20% of patients with pheochromocytomas. Of these patients, around 10% experience atrial fibrillation. Arrhythmias in the setting of pheochromocytomas tend to be difficult to control with standard β-adrenoceptor blocking agents but typically do not re-occur after the surgical removal of the tumor [8]. No large-scale studies have been published on the ideal management of pheochromocytoma due to the rarity of the disease; however, management strategies have been described based on the known physiology of pheochromocytomas and published case reports. Supraventricular tachyarrhythmias occur largely due to adrenergic effects on the atrioventricular (AV) node, leading to increased AV conduction. Reducing this conduction through the use of β-adrenoceptor blocking agents is therefore important in treating pheochromocytoma-induced supraventricular arrhythmias; however, nonselective β-adrenoceptor blockade can precipitate hypertensive crises due to the blockade of β2-adrenoceptor-mediated vasodilation, which leads to unopposed α-adrenoceptor-mediated vasoconstriction [8]. Treating patients with α-adrenoceptor blocking agents before β-adrenoceptor blocking agents may prevent this complication.

## Conclusions

This case illustrates the importance of a high clinical index of suspicion for a pheochromocytoma since it can have a variety of clinical presentations and can result in unnecessary procedures. This patient received an ablation for atrial fibrillation presumed secondary to atrial electrical abnormalities/atrial structural abnormalities. It was only after the initiation of the β-blocker post-ablation that a pheochromocytoma was included in the differential.

The case highlights the importance of appropriate α-blockade prior to the administration of β-blocker therapy, as the administration of β-blocker monotherapy after his ablation may have contributed to his hypertensive crisis and subsequent hospital admission. Atrial fibrillation in a young patient without other risk factors should prompt consideration of a pheochromocytoma.

## References

[1] Bravo EL (1994). Evolving concepts in the pathophysiology, diagnosis, and treatment of pheochromocytoma. Endocr Rev.

[2] Kassim TA, Clarke DD, Mai VQ, Clyde PW, Mohamed Shakir KM (2008). Catecholamine-induced cardiomyopathy. Endocr Pract.

[3] Manger WM, Gifford RW (2002). Pheochromocytoma. J Clin Hypertens (Greenwich).

[4] Stein PP, Black HR (1991). A simplified diagnostic approach to pheochromocytoma. A review of the literature and report of one institution's experience. Medicine (Baltimore).

[5] Motta-Ramirez GA, Remer EM, Herts BR, Gill IS, Hamrahian AH (2005). Comparison of CT findings in symptomatic and incidentally discovered pheochromocytomas. AJR Am J Roentgenol.

[6] Oshmyansky AR, Mahammedi A, Dackiw A, Ball DW, Schulick RD, Zeiger MA, Siegelman SS (2013). Serendipity in the diagnosis of pheochromocytoma. J Comput Assist Tomogr.

[7] Young WF Jr, Maddox DE (1995). Spells: in search of a cause. Mayo Clin Proc.

[8] Nazari MA, Rosenblum JS, Haigney MC, Rosing DR, Pacak K (2020). Pathophysiology and acute management of tachyarrhythmias in pheochromocytoma: JACC review topic of the week. J Am Coll Cardiol.

[9] Ferreira VM, Marcelino M, Piechnik SK (2016). Pheochromocytoma is characterized by catecholamine-mediated myocarditis, focal and diffuse myocardial fibrosis, and myocardial dysfunction. J Am Coll Cardiol.

[10] Shah NH, Ruan DT (2014). Pheochromocytoma: a devious opponent in a game of hide-and-seek. Circulation.

